# Supplementary material for: Expansion of DNA-Encoded Library Hits Using Generative Chemistry and Ultra-Large Compound Catalogs
Source: bioRxiv. 2025 Oct 2:2025.09.30.679600. Preprint. [Version 1] doi: 10.1101/2025.09.30.679600 (PMC12621893; doi:10.1101/2025.09.30.679600)
Supplement: 1 [file NIHPP2025.09.30.679600V1-supplement-1.pdf]

bioRxiv preprint doi: https://doi.org/10.1101/2025.09.30.679600; this version posted October 3, 2025. The copyright holder for this preprint (which was not certified by peer review) is the author/funder, who has granted bioRxiv a license to display the preprint in perpetuity. It is made available under aCC-BY 4.0 International license.

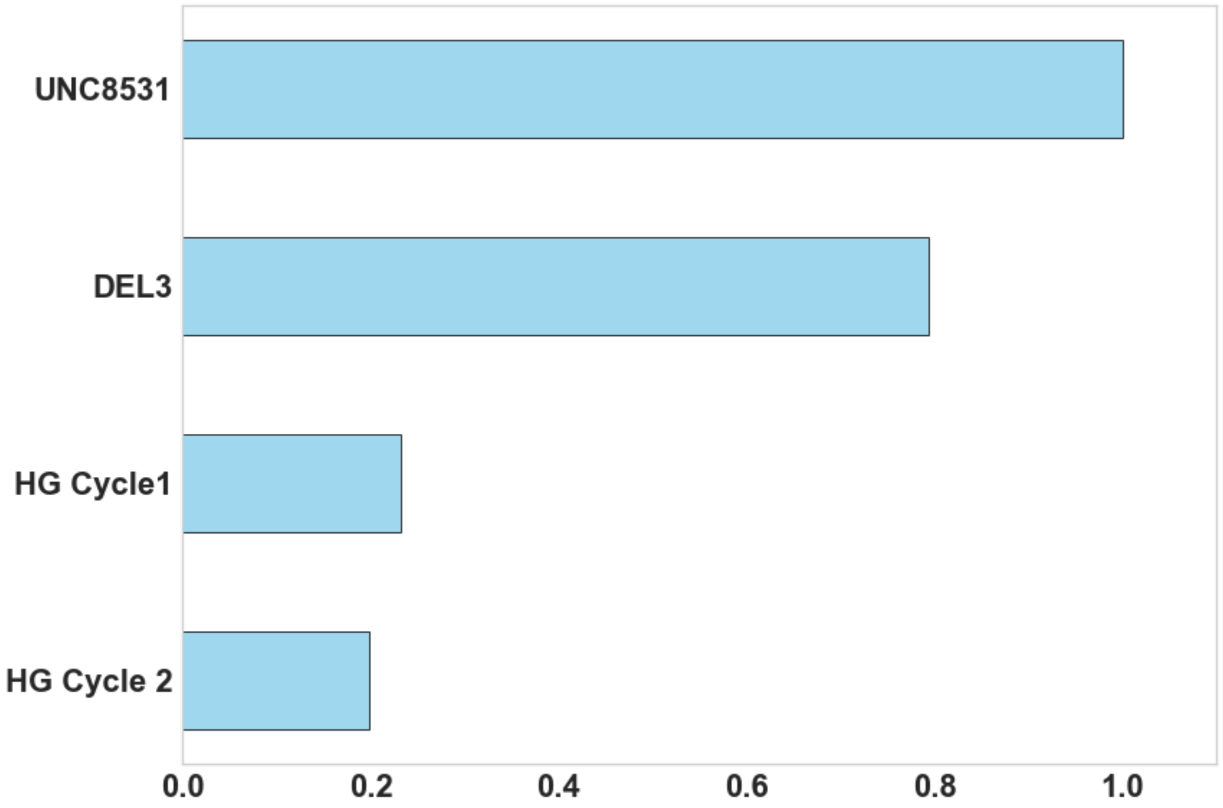

Figure S1. Visualization of the most chemically related hits from all validated actives across methods. Tanimoto similarity using Morgan fingerprints (radius=3, 2048 bits) of each compound to the original UNCDEL003 top hit, UNC8531, showing that the generative model produces the most chemically distinct active compounds across all candidates.

bioRxiv preprint doi: https://doi.org/10.1101/2025.09.30.679600; this version posted October 3, 2025. The copyright holder for this preprint (which was not certified by peer review) is the author/funder, who has granted bioRxiv a license to display the preprint in perpetuity. It is made available under aCC-BY 4.0 International license.

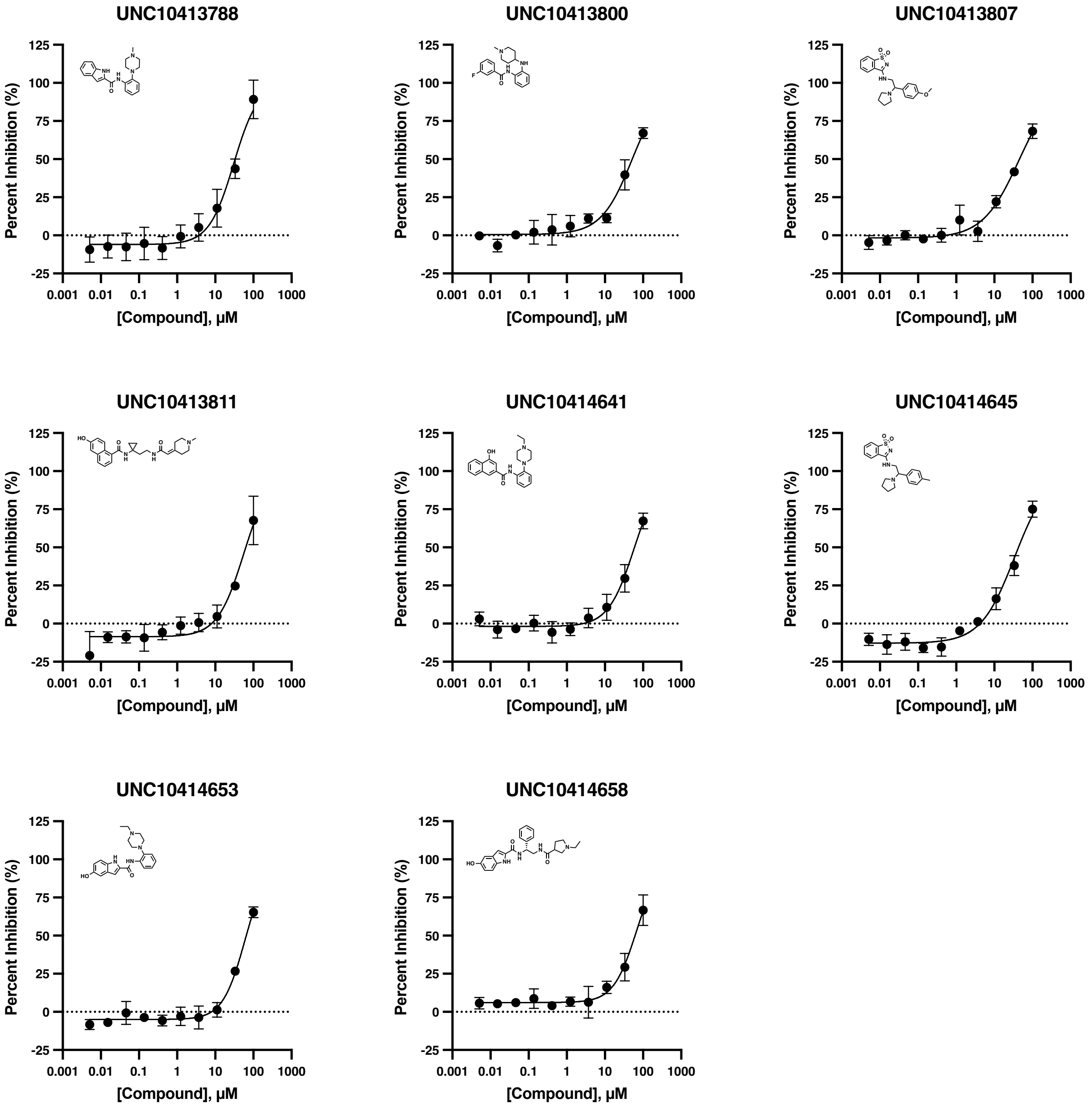

Figure S2. 53BP1 TR-FRET displacement assay dose-response curves for compounds identified from HIDDEN GEM. Each data point represents the mean of three replicates (n=3), with error bars indicating standard deviation. Dose-response curves were fitted using a four-parameter Hill equation.

bioRxiv preprint doi: https://doi.org/10.1101/2025.09.30.679600; this version posted October 3, 2025. The copyright holder for this preprint (which was not certified by peer review) is the author/funder, who has granted bioRxiv a license to display the preprint in perpetuity. It is made available under aCC-BY 4.0 International license.

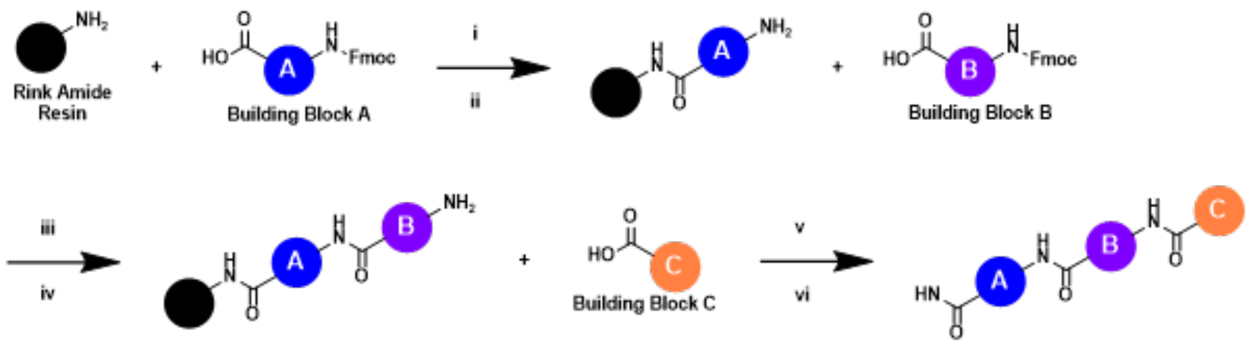

Figure S3. UNCDEL003 Compound Synthesis.
Reagents and conditions: i) Solid phase synthesis on Rink Amide resin, 3'-((((9H-fluoren-9-yl)methoxy)carbonyl)amino)-[1,1'-biphenyl]-4-carboxylic acid, HATU, DIPEA, NMP or DMF, rt; ii) Fmoc deprotection, 25% piperidine in NMP, rt; iii) Amide coupling with building block B, HATU, DIPEA, NMP or DMF, rt; iv) Fmoc deprotection, 25% piperidine in NMP, rt; v) Amide coupling with building block C, HATU, DIPEA, NMP or DMF, rt; vi) Cleavage from Rink Amide resin, 95% v/v TFA, 2.5% v/v TIPS, and 2.5% v/v H_2O, rt

bioRxiv preprint doi: https://doi.org/10.1101/2025.09.30.679600; this version posted October 3, 2025. The copyright holder for this preprint (which was not certified by peer review) is the author/funder, who has granted bioRxiv a license to display the preprint in perpetuity. It is made available under aCC-BY 4.0 International license.

Table S1. Top 10 DEL Compounds Based on Aggregated Compound Frequency

A Synthon	B Synthon	C Synthon	Compound ID	Aggregated Compound Frequency
A12	B22	C6	A12-B22-C6	82
A10	B22	C6	A10-B22-C6	74
A8	B22	C6	A8-B22-C6	66
A30	B25	C6	A30-B25-C6	62
A6	B20	C6	A6-B20-C6	61
A2	B20	C6	A2-B20-C6	60
A7	B22	C6	A7-B22-C6	56
A26	B39	C6	A26-B39-C6	55
A26	B28	C6	A26-B28-C6	54
A14	B22	C6	A14-B22-C6	53
